# Prediction of the development of metabolic syndrome by the Markov model based on a longitudinal study in Dalian City

**DOI:** 10.1186/s12889-018-5599-y

**Published:** 2018-06-07

**Authors:** Xiao Tang, Qigui Liu

**Affiliations:** School of Public Health, Dalian Medical University, No. 9 West Section Lvshun South Road, Dalian, 116044 Liaoning Province China

**Keywords:** Metabolic syndrome, Prediction, Intervention, Obesity

## Abstract

**Background:**

Metabolic syndrome (MetS) increases the incidence of cardiovascular disease and diabetes mellitus. It is essential to study the natural progression of MetS in the interest of prevention. Information on the dynamic changes in MetS in developing countries is limited. This study aimed to simulate the progression of each component of MetS and explore the potential role of these components in early prevention and intervention.

**Methods:**

This study involved 5881 individuals, aged 20 to 60 at study entry, who underwent at least two consecutive years of health check-ups in the seven-year study period at our institution’s health check-up center. Participants were divided into four groups by age (a 20- to- 40-year-old group and a 40- to 60-year-old group) and gender. A Markov model containing 7 stages (no components, isolated hypertension, isolated obesity, isolated hyperglycemia, isolated dyslipidemia, a 2-component state, and the MetS state) was constructed for each group.

**Results:**

In women and young men (20- to 40-year-old men), dyslipidemia and obesity were the two most probable states for individuals who were transitioning from no components to one of the other six states. Among those who had no components and were 30 years old at study entry, MetS was estimated to develop within 10 years in 11.42% of men and 3.04% of women. Among those who had no components and were 50 years old at study entry, MetS was estimated to develop within 10 years in 25.04% of men and 7.09% of women. The estimated prevalence of MetS over the next 10 years was higher in individuals starting with the obesity component than in individuals starting with any other isolated component. In a comparison of interventions targeting single components, simulations showed that the obesity intervention produced the largest relative reduction in the prevalence of MetS.

**Conclusion:**

Markov models are suitable for describing and predicting the dynamic development of MetS. The occurrence of MetS most frequently began with dyslipidemia or obesity. Obesity played a predominant role in the development of MetS. Early obesity intervention was extremely important for MetS prevention.

**Electronic supplementary material:**

The online version of this article (10.1186/s12889-018-5599-y) contains supplementary material, which is available to authorized users.

## Background

Metabolic syndrome (MetS) is a cluster composed of hypertension, obesity, insulin resistance, disturbed glucose and dyslipidemia. MetS increases the occurrence of cardiovascular disease (CVD) and diabetes mellitus, which result in a considerable economic burden [1, 2]. Published reports have also shown that MetS is associated closely with a variety of diseases such as acute pancreatitis, eye disease, nonalcoholic steatohepatitis and some cancers [3–6]. Other possible negative health outcomes of MetS include stress and depression [7, 8]. The prevalence of MetS in Western countries ranges from 24.0 to 38.0% [9, 10]. Surveys in Guangdong Province in southern China showed that the prevalence of MetS increased from 5.4% in 2002 to 21.3% in 2010 [11]. Considering the very large population of China as a whole, it follows that China has the largest MetS population in the world. MetS is increasingly prevalent and affects public health; thus, risk factors and early biomarkers of MetS have been investigated, including physical inactivity, diet, alcohol, high cholesterol, serum uric acid, white blood cell count and hemoglobin [12–15]. Effective interventions for the MetS population have also been reported. Previous studies have shown positive changes in MetS management after the implementation of various lifestyle interventions that differed in terms of duration, content, and format [16–18].

MetS is defined by the occurrence of at least three or four of its components: hypertension, obesity, hyperglycemia and dyslipidemia. There are 12 different stages and 144 transitions between states. Understanding the natural progression of MetS is very important for clarifying the role of its components in disease development and predicting its prevalence, which supports the development of prevention and intervention strategies. Little research has focused on the natural development of MetS and the transitions between different states. Markov models are a well-recognized method for simulating the natural history of diseases that show progression and regression between different stages. Haring et al. used a network-based approach to visualize MetS risk factor stages and their changes [19]. However, this study did not predict the future disease course. Hwang et al. applied a Markov model to describe the course of MetS development in Taiwan [20]. However, this research was only in a sample of young adults aged 18–45 years, and the data were limited due to a lack of measurements at intermediate time points. Chen et al. applied Markov models to describe the natural progression of MetS in the population of a city in eastern China, stratified by gender and age. However, this study focused only on components involved in the initial development of MetS [21].

The wide variation observed in the prevalence of MetS is speculated to be due to differences in population characteristics [22, 23]. Due to the diversity of food culture, dietary habits are substantially different between southern and northern China. Northerners prefer food rich in salt and grease, whereas southerners prefer light food and generally have a high intake of vegetables [24]. To date, the transitions among MetS components have not been characterized in northern China. Previous studies using Markov models to describe the progression of MetS have focused only on its onset. Furthermore, although some studies have assessed the effects of specific intervention programs, the interventions were on a relatively short time scale, spanning from 4 weeks to 2 years [8, 25, 26]. In this paper, we constructed Markov models to describe the development of MetS over a seven-year follow-up and to quantify the effects of putative factors. Moreover, the effects of early interventions, such as measures to reduce the number of individuals who develop each individual component, were estimated by simulation. Additionally, we compared these results with the case of no intervention over the long term in order to explore which component was the most effective target for intervention.

## Method

### Patients and data

This investigation was a retrospective cohort study. The data for this study consisted of health check-up data acquired from January 2010 to December 2016 at the hospitals affiliated with Dalian Medical University in Dalian City, located in northern China. The eligibility criteria for the study were at least two consecutive years of health check-ups in the seven-year study period and no history of cardiovascular disease or diabetes mellitus. Individuals visited the hospital twice in two consecutive years, and the interval between these two health check-ups was approximately 1 year. A total of 5881 participants were included in this study after subjects who did not give complete information were excluded. The data for the first year of study enrollment, regardless of year, were used as baseline data.

The incidence rates of CVD and diabetes as well as risk factors for these diseases, such as hyperglycemia and dyslipidemia, significantly differ across different age and gender groups [22]. In this study, we focused on young and middle-aged people for early detection and prevention of MetS. Thus, the data were limited to individuals aged 20–60 years. Participants were divided into four groups according to age (a 20- to 40-year-old group and a 40- to 60-year-old group) and gender.

Subjects were interviewed by a doctor, and information including family and personal history was collected. Blood pressure was measured twice in a seated position using a standard electronic sphygmomanometer on the right upper arm after at least 5 min of rest. The final blood pressure was the average of the two measurements. Weight and height were obtained from the subjects when they were barefoot and wearing light clothing. Body mass index (BMI) was calculated as weight in kilograms divided by the square of height in meters. Venous blood was taken after 12 h of overnight fasting. Biochemical measures included triglycerides (TG), cholesterol, serum glucose and high-density lipoprotein (HDL) cholesterol.

### MetS criteria

MetS was defined according to the Chinese Medical Association Diabetes Branch (CDS). The presence of MetS is indicated by three or four of the following: (1) BMI ≥25 kg/m^2^; (2) fasting blood glucose ≥6.1 mmol/L or a history of hyperglycemia; (3) blood pressure ≥140/90 mmHg or a history of hypertension; and (4) TG ≥1.7 mmol/L in either sex, HDL-C < 0.9 mmol/L in men, or HDL-C <1.0 mmol/L in women.

### Markov model

#### The concept of Markov chains

Many chronic diseases have a natural staged progression. A multistate Markov model is used to describe a process in which an individual moves through a series of states [27]. The state of the individual may be known at doctor or hospital visits at times 0, 1, 2, ⋯n, ⋯ . At time n, the individual is in state X_n_. A discrete-time Markov chain is a sequence of random variables X_0_, X_1_, ⋯X_n_⋯with the Markov property that the probability of moving to the next state depends only on the present state and not on the previous states. That is,

$$ \mathrm{P}\left({\mathrm{X}}_{\mathrm{n}+1}=\mathrm{j}|{\mathrm{X}}_0={\mathrm{x}}_0,{\mathrm{X}}_1={\mathrm{x}}_1,\cdots {\mathrm{X}}_{\mathrm{n}}=\mathrm{i}\right)=\mathrm{P}\left({\mathrm{X}}_{\mathrm{n}+1}=\mathrm{j}|{\mathrm{X}}_{\mathrm{n}}=\mathrm{i}\right). $$

p_ij_ = P(X_n + 1_ = j| X_n_ = i) is the probability of transition from state i at time n to state j at time n + 1. It is often calculated as the frequency of that transition. A transition matrix P = (p_ij_) includes all transition probabilities from time n to time n + 1. A Markov chain is frequently assumed to be time homogeneous, in which case the matrix is independent of time n.

Suppose that the disease has k states and that the transition between states satisfies the Markov property. The distribution of individuals over k states at time n is expressed as a row vector ${\mathrm{M}}^{\left(\mathrm{n}\right)}=\left({\mathrm{M}}_1^{\left(\mathrm{n}\right)},{\mathrm{M}}_2^{\left(\mathrm{n}\right)},\cdots, {\mathrm{M}}_{\mathrm{k}}^{\left(\mathrm{n}\right)}\right)$, where ${\mathrm{M}}_{\mathrm{k}}^{\left(\mathrm{n}\right)}$ is the number of individuals who are in state k at time n. Then, the distribution of individuals over states at time n + 1 is M^(n + 1)^ = M^(n)^P. The progression of disease can be simulated in this way.

#### Seven-stage Markov model

**Fig. 1:**
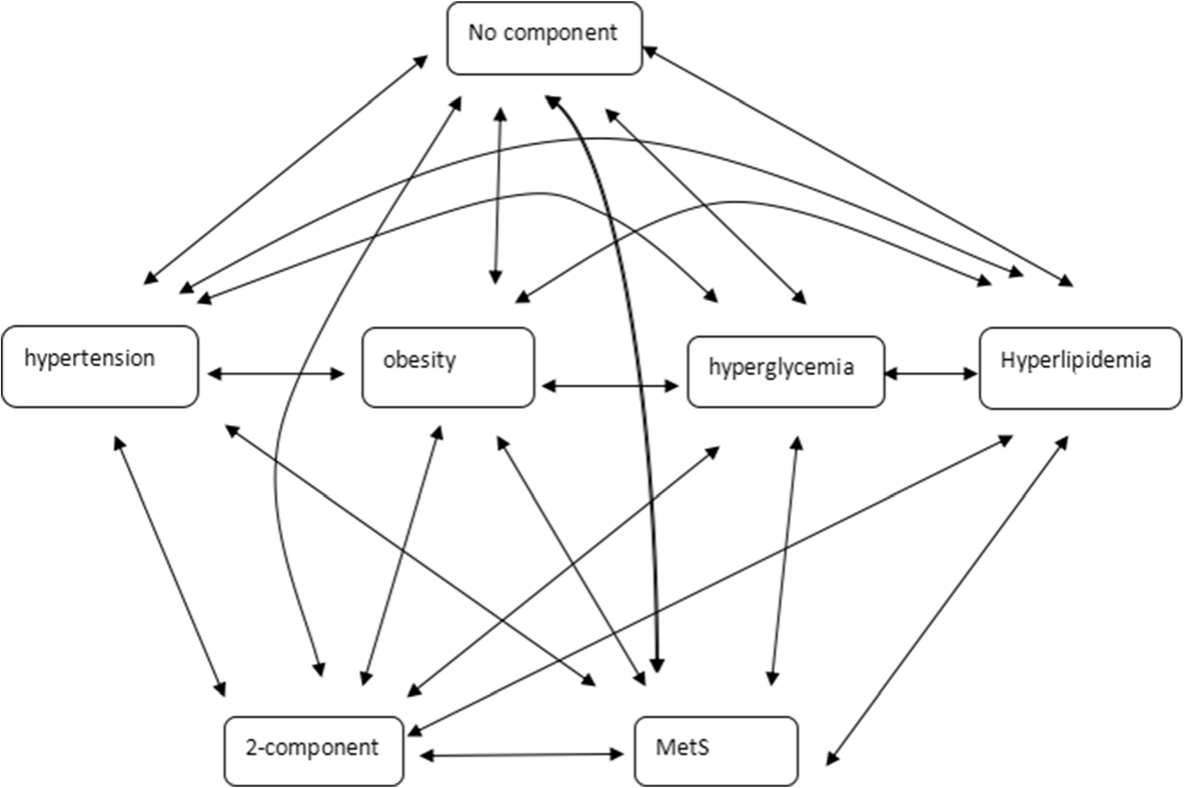
A 7-state Markov model to describe the progression of MetS components

In this study, the Markov model includes 7 states: no components, isolated hypertension, isolated obesity, isolated hyperglycemia, isolated dyslipidemia, a 2-component state, and the MetS state. The 2-component state was defined as the state in which any two isolated components occurred simultaneously. These 7 states are mutually exclusive, and there was no absorbing state. The Markov model in this study is a reversible multistate model because transitions between any two states were permitted. A map of the model is displayed in Fig. 1.

Individuals were divided into four groups by age and gender, and Markov models were constructed in each group. From health check-up data gathered over a period of 7 years, annualized probabilities of natural transitions between any two states or of the maintenance of states were obtained in each Markov model. Annual transition probabilities were first calculated as the frequency of transition from the original state to the follow-up state between each pair of consecutive years, i.e., 2010–2011, 2011–2012 and so on. The final transition probabilities were the average of these annual transition probabilities in each age and gender group. First, the individual was assumed to have no components, and the development of MetS was predicted over the next 10 years. Second, Markov models of different initial states were constructed, starting with isolated hypertension, isolated obesity, isolated hyperglycemia, isolated dyslipidemia and a 2-component state, to simulate the development of MetS over a 10-year period based on different initial states. Third, we assumed that half of the individuals diagnosed with isolated components at health check-ups were able to transition to the no-component state by diet, physical exercise or medication every year. The prevalence of MetS after 10 years was predicted under such assumptions and compared with results for the natural evolution of MetS. The relative reduction rate for MetS was calculated as the difference between the prevalence with intervention and the prevalence with natural development divided by the natural prevalence. The relative reduction rates for MetS with different isolated interventions were calculated.

The Markov models in this study satisfied two assumptions: (1) the future evolution of MetS depended only on an individual’s present state, with no dependence on previous states (the Markov chain property); and (2) transition probabilities remained constant throughout the follow-up period (time homogeneous) in each group.

### Statistical analysis

Basic data were recorded and analyzed with Excel 2007. Means and percentages were used to describe continuous and discrete variables, respectively. Matlab version 2014 was used to construct the Markov models.

## Results

General information regarding baseline and each follow-up year is presented in Table 1. A total of 5881 individuals aged 20–60 years at study entry were included in this study, and the average length of follow-up was 2.90 years. The prevalence of MetS, obesity and hypertension increased with the length of follow-up.

**Table 1:** Prevalence of components of MetS at baseline and various lengths of follow-up [n(%)]

Follow-up years						
	Baseline	1	2	3	4	5
N	5881	5881	3518	2065	1346	825
Male	2476 (42.10)	2476 (42.10)	1356 (38.54)	637 (30.85)	388 (28.83)	279 (33.82)
Hypertension	740 (12.58)	901 (15.32)	576 (16.37)	361 (17.48)	296 (21.99)	221 (26.79)
Obesity	1795 (30.52)	1942 (33.02)	1135 (32.26)	653 (31.62)	431 (32.02)	293 (35.52)
Hyperglycemia	529 (9.00)	593 (10.08)	349 (9.92)	206 (9.98)	170 (12.63)	138 (16.73)
Dyslipidemia	1278 (21.73)	1316 (22.38)	782 (22.23)	431 (20.87)	276 (20.51)	189 (22.91)
MetS	249 (4.23)	431 (7.57)	256 (7.28)	158 (7.65)	136 (10.10)	110 (13.33)

The interval between any two consecutive follow-up years was 1 year

### Markov chain model and transition probabilities

The annual transition probabilities for the Markov model, which described the natural history of MetS depending on gender and age, are presented in Tables 2, 3, 4, 5.

**Table 2:** Annual transition probabilities (%) in Markov chain models for men in the 20- to 40-year-old age group

Original state	Follow-up state						
	No components	Isolated hypertension	Isolated obesity	Isolated hyperglycemia	Isolated dyslipidemia	2 components	MetS
No components	76.40	2.86	6.72	1.29	7.30	4.72	0.71
Isolated hypertension	16.95	49.16	8.48	1.69	1.69	16.95	5.08
Isolated obesity	9.25	0.25	55.25	0.5	1.00	28.75	5.00
Isolated hyperglycemia	22.41	6.90	3.45	43.10	6.90	6.90	10.34
Isolated dyslipidemia	21.28	2.58	5.16	1.29	41.94	20.65	7.10
2 components	5.98	2.00	21.56	0.80	4.39	50.90	14.37
MetS	1.81	4.55	10.00	0	1.82	29.09	52.73

**Table 3:** Annual transition probabilities (%) in Markov chain models for men in the 40–60-year-old age group

Original state	Follow-up state						
	No components	Isolated hypertension	Isolated obesity	Isolated hyperglycemia	Isolated dyslipidemia	2 components	MetS
No components	67.63	6.81	5.79	2.04	11.24	5.46	1.03
Isolated hypertension	13.11	48.99	4.84	2.42	2.42	21.77	6.45
Isolated obesity	6.94	0.83	55.57	0.83	0.83	27.78	7.22
Isolated hyperglycemia	12.16	1.35	1.35	52.71	1.35	18.92	12.16
Isolated dyslipidemia	25.37	3.41	3.42	1.46	43.41	15.61	7.32
2 components	2.81	3.07	13.04	2.05	4.22	51.79	23.02
MetS	2.06	2.57	2.32	0.52	0	23.2	69.33

**Table 4:** Annual transition probabilities (%) in Markov chain models for women in the 20- to 40-year-old age group

Original state	Follow-up state						
	No components	Isolated hypertension	Isolated obesity	Isolated hyperglycemia	Isolated dyslipidemia	2 components	MetS
No components	87.04	1.70	3.85	0.81	3.70	2.15	0.75
Isolated hypertension	24.26	52.97	7.92	0	3.96	8.91	1.98
Isolated obesity	25.78	1.30	54.95	0.26	2.08	12.24	3.39
Isolated hyperglycemia	23.28	1.72	0	57.76	3.45	12.07	1.72
Isolated dyslipidemia	55.11	4.89	4.89	1.33	26.67	6.67	0.44
2 components	24.68	5.11	20.85	2.55	3.83	34.47	8.51
MetS	14.29	4.76	4.76	0	4.76	20.63	50.80

**Table 5:** Annual transition probabilities (%) in Markov chain models for women in the 40- to 60-year-old age group

Original state	Follow-up state						
	No components	Isolated hypertension	Isolated obesity	Isolated hyperglycemia	Isolated dyslipidemia	2 components	MetS
No components	83.78	3.39	3.85	1.64	4.85	1.98	0.51
Isolated hypertension	13.69	61.43	1.06	2.12	1.59	17.46	2.65
Isolated obesity	13.50	0	62.26	0	1.93	18.73	3.58
Isolated hyperglycemia	20.93	3.49	1.16	51.16	2.33	17.44	3.49
Isolated dyslipidemia	40.96	1.60	3.72	2.13	37.23	13.30	1.06
2 components	8.65	11.90	14.05	3.78	4.86	45.41	11.35
MetS	2.07	2.07	4.14	1.38	0	17.93	72.41

In men under 40 years old and in women, the probabilities of transition from the no-component state to the remaining six states were highest for the obesity and dyslipidemia states. In men over 40 years old, the two most probable transitions out of the no-component state were the transitions to dyslipidemia and hypertension. These results indicated that the obesity and dyslipidemia states were the states that most frequently initiated the progression of MetS.

At any given age, the probabilities of transition from the five initial states, including the isolated obesity state, isolated hypertension state, isolated dyslipidemia state, isolated hyperglycemia state and 2-component state, to MetS were always higher in men than in women. Furthermore, the transition probabilities from the no-component state to any other abnormal state were always higher in men than in women. Transitional probabilities from the isolated hypertension state, the isolated obesity state, isolated dyslipidemia state and isolated hyperglycemia state to the no-component state were lower in men than in women, which indicated that women were more likely than men in the same age group to revert from these four isolated states to a healthy state.

The probabilities of transition from any isolated state to MetS tended to be higher in the 40–60 age group than in the under-40 age group for both men and women. The probability of transition from any isolated state or the 2-component state to the no-component state generally decreased significantly with increasing age. This finding demonstrated that reversion from any isolated state or the 2-component state to the no-component state becomes more difficult with increasing age in both men and women.

Moreover, in men and in women over 40 years old, the probability of transition from the isolated obesity state to the no-component state was always lower than the transitional probabilities from the other three isolated states to the no-component state, which indicated that it is more difficult for men and middle-aged women to revert from the isolated obesity state than from any other isolated state.

### MetS development beginning with no components, any isolated component, or 2 components

**Fig. 2:**
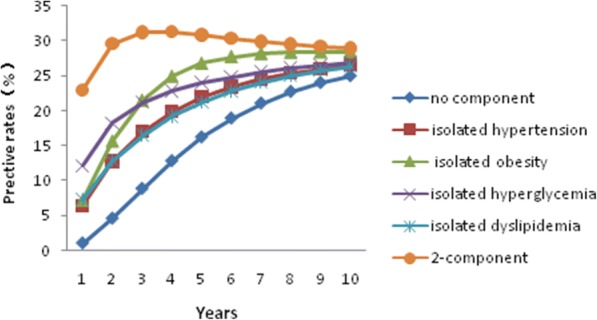
The predicted development of MetS starting with different components in 40– to 60-year-old men

**Fig. 3:**
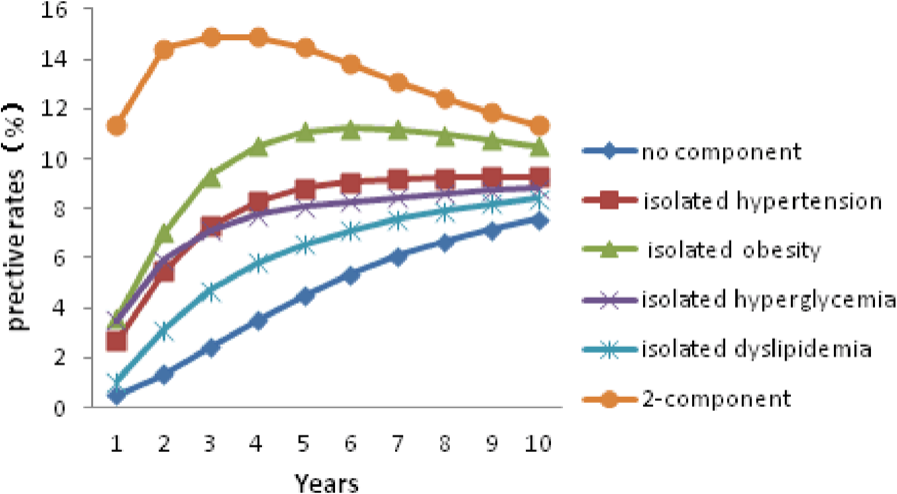
The predicted development of MetS starting with different components in 40– to 60-year-old women

The results for progression from the no-component state, any isolated component state and the 2-component state, subdivided by age and gender, are presented in Figs. 2 and 3 and in Additional files 1 and 2: Figures S1 and S2.

In each group, individuals starting with the 2-component state or any isolated component were more likely to develop MetS over the next 10 years than individuals starting with no components. The rate of MetS in individuals beginning with no components increased with time in each group. Predicted rates of MetS varied greatly between groups. After 10 years, MetS had developed in an estimated 3.04% of young women and in 11.42% of young men who had started from the no-component state at the age of 30 years. After 10 years, the estimated prevalence of MetS for men and women who were 50 years old at study entry and started with no components was 25.04 and 7.09%, respectively. Individuals who started with the obesity component were more likely to develop MetS in the next 10 years than those starting with any other isolated component.

### MetS development beginning with no components under the intervention assumption

In this step, we assume that if an individual was diagnosed with an isolated component at check-up, he/she was informed and educated sufficiently. We also assume that half of individuals would change from the isolated abnormal state to the healthy state through diet, physical training or health services every year. Under this assumption, the prevalence of MetS after 10 years for individuals beginning with no components was predicted and compared to the prevalence without intervention. The results are displayed in Table 6. All interventions aimed at isolated components were able to reduce the prevalence of MetS over a 10-year period. The relative reduction in the prevalence of MetS with the hypertension intervention or the hyperglycemia intervention was higher in middle-aged group than in the young group in both men and women. However, the relative reduction in the prevalence of MetS with obesity intervention was higher in the young group than in the middle-aged group. Almost all isolated interventions produced a higher relative reduction in the prevalence of MetS in men than in women within each age group. The relative reduction in the prevalence of MetS was highest with the obesity intervention. The isolated obesity intervention was the most effective intervention. The relative prevalence was significantly reduced if four interventions were performed simultaneously.

**Table 6:** Relative reduction in the prevalence of MetS after 10 years with intervention relative to no intervention (%)

	30 years old		50 years old	
Intervention items	Men	Women	Men	Women
Hypertension intervention	4.02	3.83	11.18	6.48
Obesity intervention	19.23	12.14	14.78	11.53
Hyperglycemia intervention	1.45	1.60	4.72	3.03
Dyslipidemia intervention	8.29	2.24	5.59	6.29
Combined intervention	32.22	18.85	34.78	27.9

Each component intervention was a hypothetical intervention such that half of individuals with an isolated component at check-up would change from the isolated abnormal state to the healthy state every year

### Validation of the model

Health check-up data acquired from another cohort study from 2014 to 2017, which involved 2248 individuals aged 20 to 60 years, were used to validate our model. Health check-up data were collected each year, beginning in 2014. The estimated prevalence rates of MetS after 3 years were 14.8% (20- to 40-year-old men), 33.1% (40- to 60-year-old men), 3.4% (20- to 40-year-old women) and 11.7% (40- to 60-year-old women). The actual prevalence rates of MetS within the cohort in 2017 were 14.0, 34.8, 2.9, and 11.8%, respectively, for the four aforementioned demographic groups. The estimated prevalence of MetS was similar to the prevalence in this cohort.

## Discussion

MetS is closely associated with its components, and its progression includes several states among which individuals transition randomly. Although much research exists on MetS, most research has ignored the complexity of the disease and the reversal of MetS-associated states. In describing the natural history of a disease and identifying the predominant risk component, modeling its progression is more accurate than presenting simple incident rates [28]. Markov models are suitable for representing dynamic changes between states and predicting the prevalence of a disease.

Existing publications concerning gender differences in the prevalence of MetS are not consistent; some studies report a higher prevalence of MetS in men than in women [29, 30], while others report the opposite [31]. In this cohort, men were more prone to developing MetS than women were and had a higher predicted prevalence after 10 years. We found some evidence in the current sample that the probabilities of transition from any isolated abnormal state to the 2-component state or to the MetS state were higher in men than in women, and the probabilities of transition from any isolated abnormal state to the no-component state were lower in men than in women. These two facts indicated that men with abnormal components were more likely to develop MetS and were more resistant to transitioning to the healthy state than women with the same components.

In the current study, age was an important contributor to MetS. The middle-aged population was more likely than the young population to develop MetS. A longitudinal study of 17,014 subjects in the municipality of Tromso showed that MetS risk significantly increased with age in women but not in men [29]. Chiodo et al. observed a higher prevalence of MetS in the elderly than in the middle-aged [32]. Chedraui et al. demonstrated that IL-6 and urokinase-type plasminogen activator levels differed between postmenopausal women with MetS and other women; this difference was associated mainly with metabolic abnormalities [33].

It is still disputable which component of MetS is likely to occur first in the progression. Hwang et al. claimed that the primary initiating components in young men were hypertension and the 2-component state, while in young women, the primary initiating states were obesity and low HDL [20]. Chen et al. found that obesity and dyslipidemia were the most likely components in men under 60 years old and women under 50 years old, while the 2-component state and hypertension were the most likely states (besides those described above) in the elderly population [21]. Our data indicated that obesity and dyslipidemia were the components most likely to initiate the development of MetS in women, regardless of whether they were young or middle-aged. Obesity and dyslipidemia were also the main initiators in young men. However, in middle-aged men, hypertension was the most important initiator aside from dyslipidemia and obesity.

Our study demonstrated that obesity plays an important role in the development of MetS. First, in the current cohort, obesity was an important component in the initiation of MetS in young and middle-aged populations. Second, among men and middle-aged women, individuals with obesity were more resistant to reverting from the isolated obesity state to the no-component state than those with other isolated states. This result was partly consistent with a previous report by Okada et al., who claimed that obesity was particularly serious and more resistant to reversal than the other four components of MetS [34]. Third, the highest prevalence of MetS appeared in individuals starting with the obesity state when we predicted the development of MetS over the next 10 years; this prevalence was higher than that for individuals who started with different isolated components. This result indicated that obesity was the best predictor of the development of MetS. Chen et al. conducted a 10-year prediction study with the population of Dongying City in China; the authors claimed that individuals with isolated dyslipidemia had a greater chance of developing MetS than those with any other isolated state [21]. This result was not consistent with the results of the current study. Lastly, our data showed that obesity control reducing the transition probability from no components to obesity was more effective than control of any other isolated component in reducing the prevalence of MetS. Wiria et al. performed a cross-sectional study in Flores, Indonesia, and found that helminth infections were negatively associated with risks of MetS, such as BMI and serum cholesterol levels; the authors claimed that this association was partially mediated by an effect on BMI [35]. This result indirectly demonstrated that obesity control can effectively reduce the risk of MetS.

The question of which component is the principal factor contributing to MetS remains controversial. Some studies have reported that hypertension is the risk factor most strongly associated with MetS diagnosis [25, 36]. Other research has proposed that obesity plays a central role in the development of MetS or precedes other MetS components. Haring et al., using data from a five-year follow-up of a longitudinal cohort, identified central obesity as the predominant risk factor cluster [19]. Cameron et al. conducted longitudinal surveys over 5 years and claimed that central obesity was the dominant risk factor predicting deterioration of other MetS components [37]. Further support for the role of central obesity in the pathogenesis of MetS has come from physiology. The pathophysiology of obesity is related to a diet containing excess calories and/or high saturated fat or glucose content. When nutrient intake exceeds the metabolic demand for energy, TG are stored in adipocytes, which release adipocytokines that regulate the components of MetS, including insulin sensitivity and blood pressure as well as glucose and lipid metabolism [14].

MetS increases the occurrence of cardiovascular disease (CVD) and diabetes mellitus, but people with MetS are often unaware of their condition because of the subtle and nonspecific symptoms. Therefore, it is critical to implement interventions in persons with MetS. Lifestyle intervention programs implemented in Hong Kong, China, have shown a positive effect in reducing the risk of MetS [38]. If people with isolated components appreciate the consequences of MetS and change their lifestyle, they may find it less difficult to change their state than those with MetS, considering the lower economic burden and earlier disease state. Predictions based on our data showed that interventions in each of the isolated components could reduce the prevalence of MetS. Weight control was promoted in each group because this intervention was associated with the largest decline in MetS prevalence among all the isolated interventions. Blood pressure control was important in the middle-aged group, and lipid control was effective in men and in middle-aged women. Education has been shown to improve motivation to adhere to a healthy lifestyle [39]. Therefore, there is still much work that needs to be done to motivate and educate people affected by these isolated components. Early intervention aimed at people with these isolated components is advised.

This study showed that obesity played a predominant role in the development of MetS, and weight control was the focus of intervention at the place and time of the study. However, the characteristics of MetS development varied greatly across different populations and time scales [19–21]. With economic development and the improvement of people’s health consciousness, the roles of MetS components will change. Transition probabilities and Markov models can help to clarify the role of MetS components and adjust the focus of prevention and intervention over time.

This study had some limitations. First, the “natural” transition probabilities may be not completely natural because some individuals received interventions through health services. Second, there was no absorbing state in the model because we did not consider mortality. Mortality data for this cohort were unavailable. However, mortality is low in the general population receiving health examinations; therefore, we assumed that the influence of mortality in the model was very small. Third, the effects of all interventions in this study were estimated under the assumptions we have described. However, we used simulations to identify the most effective intervention, providing theoretical support for the future implementation of the intervention.

## Conclusions

Markov models are suitable for describing and predicting the dynamic development of MetS. In women and young men (20- to 40-year-old men), the development of MetS began principally with dyslipidemia or obesity. In middle-aged men, hypertension became the important initiator aside from dyslipidemia. Obesity played a predominant role in the development of MetS. Early obesity intervention was extremely important for MetS prevention.

## Additional files

Additional file 1: **Figure S1.** The predicted development of MetS starting with different components in 20– to 40-year-old men. (TIF 543 kb)

Additional file 2: **Figure S2.** The predicted development of MetS starting with different components in 20– to 40-year-old women. (TIF 543 kb)
