# Supplementary figures and images for: Prediction of the development of metabolic syndrome by the Markov model based on a longitudinal study in Dalian City
Source: BMC Public Health. 2018 Jun 7;18:707. doi: 10.1186/s12889-018-5599-y (PMC5992701; doi:10.1186/s12889-018-5599-y)

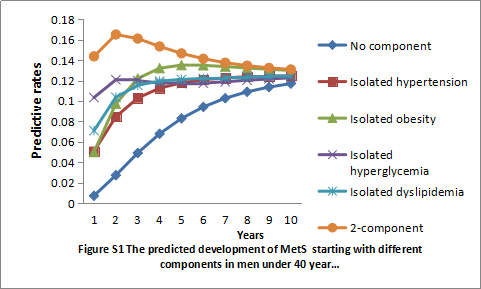

Supplement: Supplementary file 1 — Figure S1. The predicted development of MetS starting with different components in 20– to 40-year-old men. (TIF 543 kb) [file 12889_2018_5599_MOESM1_ESM.tif]

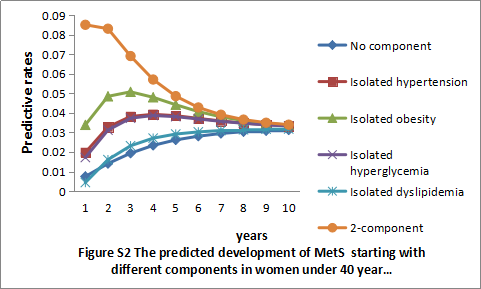

Supplement: Supplementary file 2 — Figure S2. The predicted development of MetS starting with different components in 20– to 40-year-old women. (TIF 543 kb) [file 12889_2018_5599_MOESM2_ESM.tif]
